# Huge Pyogenic Granuloma of the Penis

**DOI:** 10.1155/2015/263168

**Published:** 2015-07-02

**Authors:** Fatih Akbulut, Tugba Akbulut, Faruk Kucukdurmaz, Erkan Sonmezay, Abdulmuttalip Simsek, Gokhan Gurbuz

**Affiliations:** ^1^Department of Urology, Haseki Training and Research Hospital, 34096 Istanbul, Turkey; ^2^Department of Dermatology, Istanbul Faculty of Medicine, Istanbul, Turkey; ^3^Department of Urology, Nizip State Hospital, Gaziantep, Turkey

## Abstract

Pyogenic granulomas are benign vascular disorders of the skin and mucose membranes, generally developed by trauma and irritation. The lesions are generally small. They are most commonly seen in the skin and oral mucosa and rarely seen on penis. We present the case of a huge pyogenic granuloma on the penis.

## 1. Introduction

Pyogenic granulomas (PGs) are benign vascular lesions, which generally originate from skin and/or mucous membranes. These lesions mostly present as solitary red papules or polyps that rapidly grow in several weeks or months. Eventually, this rapid growth stops and final size of the lesions rarely exceeds 1 cm in most cases. PGs may develop at any age but are most often seen in children and young adults. They are equally common in males and females and exhibit no racial or familial predisposition. These lesions can be seen in various organs but have rarely been reported on the penis [1]. We present a patient with a huge polypoid pyogenic granuloma of the penile shaft.

## 2. Case Report

**Figure 1:**
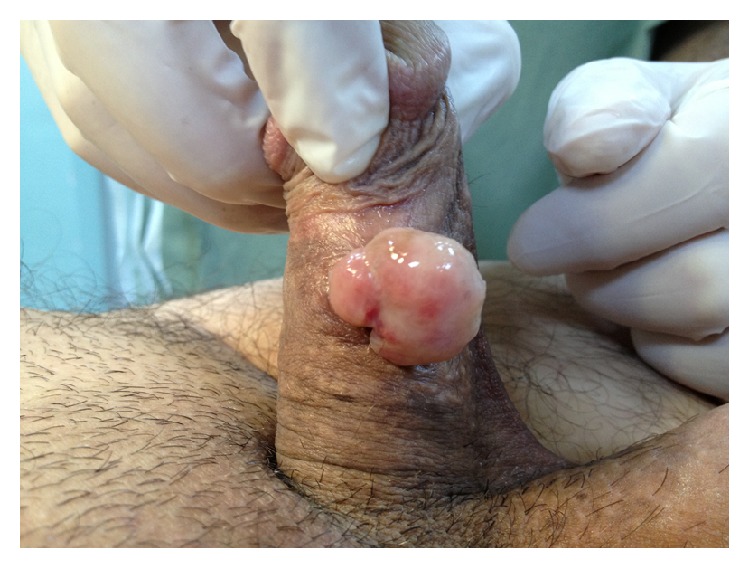
The polypoid appearance of pyogenic granuloma.

**Figure 2:**
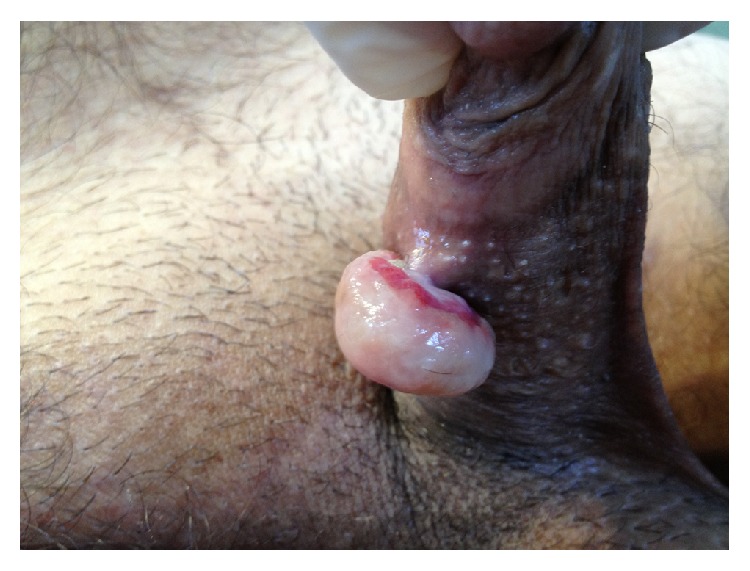
Pyogenic granuloma of the penis, which had a narrow pedicle.

A 42-year-old circumcised man was admitted to an outpatient clinic of dermatology with a 3-month history of a lesion on the penile shaft. The patient did not suffer from pain, bleeding, or leakage and the lesion went on growing during the process. After dermatological evaluation, the patient has been referred to our department for excisional biopsy. The size of the lesion was 4 × 2 cm and it had a polypoid appearance and fragile surface (Figure 1). The lesion had a narrow pedicle on the right lateral part of the penile shaft (Figure 2). We referred the patient to a dermatology clinic in a university hospital for a second advice. The presumptive diagnosis of the dermatologist was pyogenic granuloma and she also recommended excisional biopsy for diagnosis and treatment. The lesion was excised under local anesthesia. During the operation, it was observed that the pedicle had a well-demarcated vascularized area and it was ligated at the base of the lesion. The diagnosis of pyogenic granuloma was histologically confirmed by pathological evaluation. During the postoperative follow-up, there was no recurrence 4 months after surgery.

## 3. Discussion

Pyogenic granulomas are rapidly growing, red, friable, papular, or polypoid lesions of the skin or mucosa that frequently ulcerate and are most commonly seen in children and young adults. The histological pattern of these lesions consists of lobules of small capillaries set in a fibromyxoid matrix, often distinctly exophytic and bounded by hyperplastic epithelium. PGs are noninfectious and purulent lesions, which are thought to develop as reactive inflammatory masses of blood vessels and a few fibroblasts within the dermis of the skin [2]. The etiology is not fully understood. Rapid growth occurs in response to an unknown stimulus that triggers endothelial proliferation and angiogenesis. Trauma and irritation can provoke the sequence, but generally there is no identifiable cause. In our case, there was no explainable reason for it.

Pyogenic granulomas can be seen in various locations of the body. They are commonly found on the hands, fingers, arms, face, and neck. PGs of penis have been rarely reported [1, 3]. Also, the diameter of PGs is rarely greater than 1 cm. To our knowledge, there is only one case of a huge (7 × 4 cm) PG developed on the glans penis of a 29-year-old male [1]. The lesion started as a red papule 2 years ago, and it had been growing until he applied the clinic. The process of this patient's history was similar to our case.

There are various treatment options for PGs: surgical excision, electrodissection, cryotherapy, sclerotherapy, curettage/shave excision, lasers, imiquimod cream, and microembolization. Since spontaneous involution of PG has been rarely reported, it would mostly require one of these treatment options due to aesthetical reasons [4]. Surgical excision and primary closure were found to be associated with low recurrence rates among surgical treatments. Cryotherapy had low recurrence risk among nonsurgical treatment options of PG. There was no statistically significant difference between treatment by surgical excision and cryotherapy [5]. Additional advantages of surgical treatment are single step treatment and pathological evaluation. Pathological confirmation is beneficial for follow-up. Although clinical characteristics and history are very often adequate to distinguish a PG from the other lesions, up to 18% of these lesions are misdiagnosed [6]. Nonsurgical treatment options should be applied for nonsurgical candidates of PG, which are on cosmetically sensitive areas such as face or which are close to vital organs.

## 4. Conclusion

We report a huge PG growing on the shaft of the penis, treated by surgical excision and primary closure. We recommend excision of huge PGs of the penis due to its advantages like low recurrence rates, single step treatment, and the opportunity of pathological evaluation.
